# The complexity of bladder cancer: long noncoding RNAs are on the stage

**DOI:** 10.1186/1476-4598-12-101

**Published:** 2013-09-05

**Authors:** Quanan Zhang, Mo Su, Guangming Lu, Jiangdong Wang

**Affiliations:** 1 Department of Oncology, the Second Affiliated Hospital, Southeast University, Nanjing, 210009 Jiangsu, China; 2 Department of Pathology, Laboratory of Molecular Pathology and Molecular Imaging, Jinling Hospital, School of Medicine Nanjing University, 305 Zhong Shan Dong Lu, 210002 Nanjing, China

**Keywords:** Bladder cancer, Long noncoding RNAs, lncRNAs, Gene regulatory network

## Abstract

The mammalian genome encodes thousands of long noncoding RNAs (lncRNAs) and it is increasingly clear that lncRNAs are key regulators of cellular function and development. Gain and/or loss of function studies in cell culture indicate that lncRNAs can regulate gene transcription indirectly through the targeting and recruitment of chromatin-modifying complexes as well as directly at the transcriptional or posttranscriptional levels. LncRNA biology is attracting great attention in cancer research because dysregulated lncRNAs occur in a variety of cancers, placing lncRNAs on the stage of cancer genome research. We briefly describe the latest lncRNA biology and discuss the oncogenic lncRNAs involved in core pathways in bladder cancer and the application of lncRNAs to its diagnosis and targeted treatment. LncRNAs are becoming essential components of the gene regulatory circuitry in the complexity of bladder cancer.

## Introduction

Bladder cancer is the tenth most common malignancy in women and it is the fourth most common in men [1]. It comprises at least two major groups: low grade papillary tumors and high grade invasive tumors. The majority of malignant bladder tumors are urothelial cell carcinomas that evolve from the epithelial lining of the bladder wall and non-invasive papillary tumors of urothelial carcinomas that commonly recur but rarely progress. However, invasive bladder tumors are more aggressive, presenting with penetration of the basement membrane or invasion into muscle [2]. Patients with invasive disease have a much worse prognosis, with only a 50% 5-year survival [3]. Two altered molecular pathways appear to genetically explain most cases of bladder cancer: the one harbors gene mutations that constitutively activate the receptor tyrosine kinase-Ras pathway, the other involves deficits in TP53 and/or *RB* tumor-suppressors [2]. Mutations in these two molecular pathways of tumor development usually predict outcome of the malignancy.

Genome-wide studies of expression profiling and discovery of small non-coding RNA affecting gene expression have dramatically changed what was a simple classification of bladder cancer pathogenesis into two alternative molecular pathways. Altered gene expression in bladder cancer, with both up- and down-regulation, may involve up to 500 protein coding sequences for low-grade non-invasive tumors and up to 2300 genes for high-grade invasive tumors [4]. Moreover, in many clinical cases, mutations were not found inside the coding sequences of genes in the two molecular pathways, but the expressions of these genes were changed, indicating that epigenetic modifications may play an important role in tumor development. Indeed, several genome-wide methylation assessments in these neoplastic tissues have been published, and an increasing number of small non-coding RNAs are either up-regulated or down-regulated in bladder cancer, indicating that impaired gene expression may also occur in these molecular pathways [5]. Recent studies have demonstrated that long non-coding RNAs (lncRNAs) play important roles in carcinogenesis and cancer metastasis [6-8] and aberrant expression of lncRNAs has been identified in bladder cancer. LncRNAs may function as oncogenes or tumor suppressors in the cancer initiatome [9] and, therefore, bladder cancer can no longer be considered as a simple model of malignancy.

In the present review, we summarize recent progress in the genome-wide analysis of lncRNAs in bladder cancer and the dysregulation of lncRNAs in bladder cancer tissues or cells. We delineate the regulatory network mediated by lncRNAs and the implication of lncRNAs for diagnosis, assessment and treatment of bladder cancer. We suggest that lncNRAs add a new, but informative layer to our understanding of the complexity of bladder cancer development.

### LncRNAs and their functions

**Figure 1:**
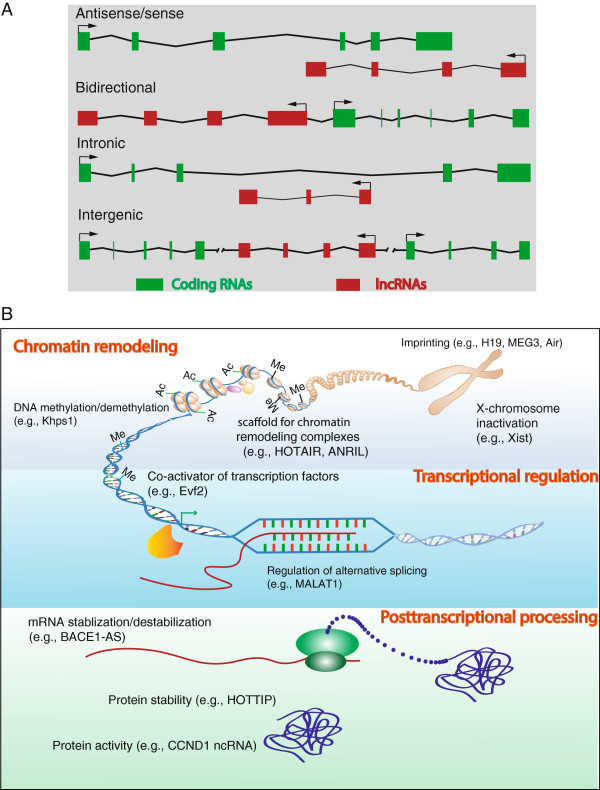
**Genomic structures and functions of long noncoding RNAs (lncRNAs). (A)** Classification of lncRNAs according to their genomic locations relative to nearby protein coding genes: Antisense lncRNA— transcribed in the opposite direction of coding genes, and overlapped with a coding exon(s); Bidirectional lncRNA—transcribed from the promoter of a protein-coding gene and in opposite direction and, in general, within a few hundred base pairs; Intronic lncRNA—transcribed from inside of an intron of a protein-coding gene; Intergenic lncRNA—transcribed from between two protein-coding genes separated by a distance of five kilo base pairs. **(B)** Mechanisms of lncRNA function: lncRNAs regulate gene expression in a cis or trans manner via recruitment of proteins or molecular complexes to specific loci, scaffolding of protein complexes, titration of RNA-binding factors or as decoys, allowing other RNAs to start posttranslational regulation.

LncRNAs are non-protein coding transcripts longer than 200 nucleotides. This arbitrary limit of length distinguishes lncRNAs from small regulatory RNAs such as microRNAs, Piwi-interacting RNAs, small nucleolar RNAs and short interfering RNAs. LncRNAs can be classified into five broad categories, according to their genomic locations relative to protein coding genes: (1) sense, or (2) antisense, when overlapping one or more exons of another transcript on the same or opposite DNA strand, respectively; (3) bidirectional, when the sequence is located on the opposite strand from a neighboring coding transcript whose transcription is initiated less than 1000 base pairs away, (4) intronic, when it is derived wholly from within an intron of a second transcript, or (5) intergenic, when it lies within the genomic interval between two genes [10] (Figure 1A). So far, LNCipedia database has collected 32,183 human annotated lncRNAs [11] and NONCODE contains 73,327 published human andmouse lncRNAs [12].

New lncRNAs will continue to be discovered with the advent of the high-throughput transcriptome sequencing. A majority of lncRNAs are transcribed by RNA polymerase II, spliced and polyadenylated, while a few lncRNAs are transcribed by RNA polymerase III [11]. In general, lncRNAs are less conserved than protein coding genes and exhibit tissue-specific and cell-specific expression features [13,14]. Additionally, most lncRNAs are located in either the cytoplasm or the nucleus, although some are found in both cytoplasm and nucleus [14].

LncRNAs have diverse functions in different physiological and pathological states. They may participate in global cellular behaviors by controlling apoptosis, cell death and cell growth [15]. They may also be key regulators of biological processes, including stem cell pluripotency and neurogenesis [16,17] and cell differentiation [18]. LncRNAs regulate gene expression at various levels, including chromatin modification [8], transcription, and posttranscriptional processing [19] and these are illustrated conceptually in Figure 1B.

LncRNAs play classic roles in imprinted gene expression. Diploid organisms carry two alleles of genes, one from each parent’s autosomes. In most cases, both alleles are expressed equally, except when a subset of genes shows imprinting and, in that case, expression is restricted by an epigenetic mechanism to either the maternal or paternal allele. H19 and *Xist* (X inactivated specific transcript) are imprinted lncRNAs that were identified in the early 1990s [20,21]. H19 is an autosomal lncRNA that is expressed on the maternally derived autosomal chromosome and it maintains silencing of the *IGF2* gene on that chromosome, thereby allowing expression of only the paternally derived *IGF2* gene [20]. X-chromosome inactivation (XCI) is effectively a dosage-compensation process that equalizes expression of X-chromosomal genes between males and females by inactivating one of the two X chromosomes in female cells. The process of XCI is regulated by the Xist lncRNA that interacts with polycomb repressive complex 2 (PRC2) and propagates epigenetic silencing of an individual X chromosome [22]. The core of PRC2 comprises EZH2 (enhancer of zeste homolog 2), SUZ12 (suppressor of zeste 12) and EED (embryonic ectoderm development). EZH2 functions as a histone H3 Lys 27 (H3K27) methyltransferase. Trimethylation of H3K27 (H3K27me3) correlates with a transcriptionally-repressed chromatin state. Indeed, many human lncRNAs associate with chromatin-modifying complexes that include PRC2 and lysine specific demethylase 1/REST co-repressor 1/RE1-silencing transcription factor (LSD1/CoREST/REST) protein complexes, and the result is a suppression of gene expression by the complexity of epigenetic regulation. This mechanism of action of lncRNAs is seen in several examples.

Biochemical experiments showed that the 5′ domain of HOTAIR (HOX antisense intergenic RNA) lncRNA binds to EZH2, whereas the 3′ domain of HOTAIR binds to LSD1/Co-REST/REST complex. Thus, HOTAIR serves as a scaffold to assemble and target PRC2 and LSD1/CoREST/REST complexes to the HOXD locus and coordinates H3K27 methylation and H3K4 demethylation for transcription repression [23]. Other lncRNAs, like ANRIL (antisense non-coding RNA in the INK4 locus), may be required to recruit PRC2 to specific genomic loci, resulting in suppression of CDKN2A expression [24].

The mode of action of some lncRNAs is by an interaction with their intracellular steroid receptors, such as GAS5 (growth arrest specific 5), to regulate downstream target gene expression [25]. Other lncRNAs regulate transcription through a variety of mechanisms, including interaction with RNA-binding proteins, acting as a co-activator of transcription factors (e.g., Evf2) [26] or repressing a major promoter of their target gene [27]. In addition, lncRNAs can regulate gene expression at the posttranscriptional level such as stabilizing of specific mRNAs (e.g., BACE1-AS) [28].

### Aberrant lncRNA expression in bladder cancer

Recent studies demonstrated that lncRNAs play important roles in carcinogenesis and cancer metastasis [6,7]. We speculate that lncRNAs may also be involved in bladder cancer initiation, development and metastasis. The general strategy to find cancer-associated lncRNAs is to compare lncRNA expression profiles in bladder cancer tissue with adjacent non-neoplastic tissue, using conventional molecular biology techniques (e.g., subtractive hybridization, cDNA microarrays, and polymerase chain reaction-based methods). These “guilty by association” studies have found numerous bladder-cancer associated lncRNAs (see Table 1).

**Table 1:** List of aberrant long noncoding RNAs in bladder cancer

**lncRNA**	**Location**	**Expression**	**Methods**	**References**
H19	Chr11p15.5	Up	PCR based screening	[30,31]
MALAT-1	Chr11q13.1	Up	PCR-based	[32,33]
TUG1	Chr12	Up	qRT-PCR	[34]
UCA1 UCA1a	Chr19p13.12	Up	Subtractive Hybridization, and PCR	[35,36]
Linc-UBC1	Chr1q32.1	Up	Microarray screening and qRT-PCR	[37]
MEG3	Chr14q32.3	Down	qRT-PCR	[38]

Gain- or loss-of-function studies suggested that lncRNAs can be functionally categorized as either oncogenic lncRNAs or tumor-suppressor lncRNAs, when their expression levels changed in the cancer initiatome [9]. For example, the lncRNA Urothelial Cancer Associated-1 (UCA1) has been screened and cloned from the human bladder (transitional cell carcinoma, TCC) cell line BLZ-211 [29]. UCA1 is highly expressed in embryonic tissues, bladder cancers and other cancers, but not in adult tissues or adjacent non-neoplastic tissues, which indicates that UCA1 may be involved in both embryonic development and carcinogenesis. Furthermore, proliferation, migration, invasion, and drug resistance were increased after UCA1 was ectopically expressed in BLZ-211 bladder cell lines. When BLZ-211 cells, expressing UCA1, were inoculated into nude mice, their capacity for tumor formation was increased [29] and strongly suggested that UCA1 has oncogenic function in bladder cancer development.

The lncRNA H19 is one of the earliest-discovered noncoding RNAs in the mammalian genome. The imprinted H19 gene is highly expressed in human embryos and fetal tissues, but its expression is almost completely shut off in adults [30]. Nevertheless, H19 is re-expressed in a number of tumors, including bladder carcinoma, demonstrating that it is an onco-fetal RNA [30]. H19 expression levels were remarkably increased in bladder cancer tissue as compared with adjacent normal control tissue [31-33]. Ectopic expression of H19 promotes bladder cancer cell proliferation in vitro [31] and enhances bladder cancer cell migration, both in vitro and in vivo [32]. Therefore, H19 appears to be an onco-lncRNA and serves as a tumor marker in bladder cancer. More recently, a new lncRNA, *linc-UBC1* (Up-regulated in Bladder Cancer 1), was found to be over expressed in ~60% of invasive bladder cancer tissues and it was correlated with lymph node metastasis and poor survival [34]. *MALAT1* (Metastasis Associated Lung Adenocarcinoma Transcript 1) was originally identified to be highly expressed in metastatic small cell lung cancer [35], but recent studies showed that *MALAT1* is upregulated in bladder cancer and its expression level corresponds to the tumor grade and metastatic stage [36,37]. When they are taken together, most, if not all highly expressed lncRNAs in bladder cancer appear to be oncogenic.

On the other hand, Maternally Expressed Gene 3 (*MEG3*) is an imprinted gene that encodes a lncRNA and it is negatively associated with tumorigenesis. Various lines of evidence support a tumor suppressor function for MEG3 lncRNA. For example, *MEG3* is expressed in many normal tissues, but its expression is lost in primary human pituitary tumors and cell lines [38,39] as well as in bladder cancer [40]. Multiple mechanisms contribute to the loss of MEG3 expression in tumors, including gene deletion, promoter hypermethylation, and hypermethylation of the intergenic, differentially methylated region [32]. Re-expression of MEG3 inhibits tumor cell proliferation in culture and colony formation in soft agar and the underlying mechanism of growth inhibition is partly the result of MEG3-induced apoptosis [39].

With the advent of next generation sequencing technologies, RNA-seq has been widely used to discover novel noncoding RNA transcripts including lncRNAs in cancer [13,34,41]. Therefore, we can anticipate that more novel lncRNAs will be discovered in bladder cancer using RNA-seq technology, even though genome-wide transcriptome study of bladder cancer with this technology has not yet been reported in the literature.

### LncRNA-mediated regulatory network in bladder cancer

Alterations in the Ras-MAPK and PI3K-AKT-mTOR pathways are largely responsible for promoting cell growth in urothelial neoplasia [2]. Since lncRNAs are essential elements of the regulatory circuits and play important roles in cancer development [6], we would ask whether the dysregulated lncRNAs, as described above, regulate the key pathways in bladder cancer. Several groups have begun to address this question with compelling experiments. Yang et al. [42] knocked down UCA1 in BLZ-211 cells and found that the expressions of several cell cycle-related genes (e.g., *CDKN2B*, *EP300* and *TGFβ-2*) decreased and both the encoded p300 and its coactivator, cAMP response element-binding protein (CREB), levels were significantly down regulated. Activation of CREB and AKT is positively correlated with the expression level of UCA1. Furthermore, UCA1 regulated the cell cycle through CREB in the PI3K-AKT dependent pathway in bladder cancer [42].

**Figure 2:**
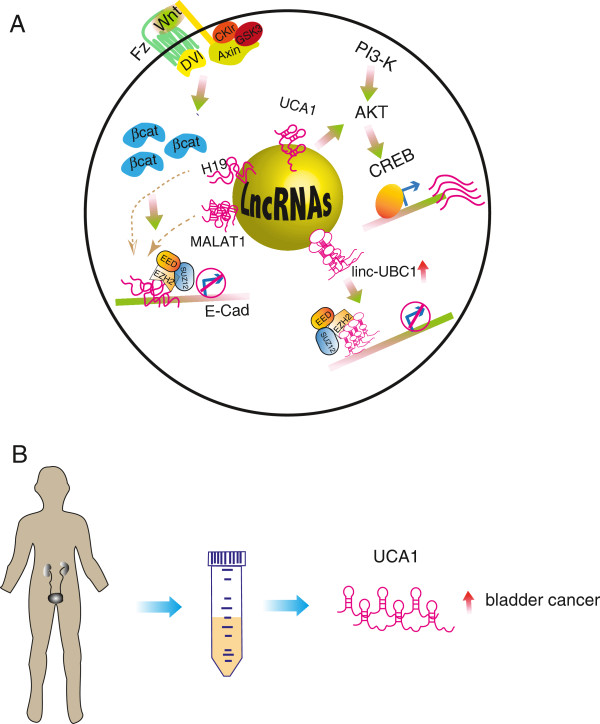
**Long noncoding RNA (lncRNA)-mediated signaling pathways in bladder cancer and its applications. (A)** Oncogenic lncRNAs activate proliferative pathways, such as PI3K-AKT and Wnt/β-catenin pathways; lncRNAs such as UCA1, H19, MALAT1 and linc-UBC1 are overexpressed in bladder cancer and epigenetically regulate gene expression in concert with core cancer pathways during tumorigenesis. **(B)** LncRNA UCA1 as a biomarker for noninvasive detection in urine.

H19 is essential for human tumor growth and metastasis through its interaction with several proteins. Over-expression of H19 resulted in a significant increase in expression of ID2 (inhibitor of DNA binding/differentiation 2), whereas a knockdown of H19 expression decreased ID2 expression [31], suggesting that up-regulated H19 increases bladder cancer growth by regulating ID2 expression. However, it is unclear whether H19 regulates ID2 expression or it is solely a correlation. Up-regulated H19 not only promotes bladder cancer cell proliferation, but it also promotes cell migration in vitro and in vivo [32]. The underlying mechanisms of H19-mediated metastasis appear to be associated with EZH2 and this association results in Wnt/β-catenin activation and subsequent down-regulation of E-cadherin. A significant negative correlation is also observed in vivo between levels of H19 and E-cadherin [32]. More experimental evidence is needed to pinpoint the detailed mechanisms of H19 mediated Wnt/β-catenin pathways. Interestingly, the Wnt signaling pathway is activated when *MALAT1* overexpression promoted epithelial-mesenchymal transition (EMT) in bladder cancer [37]. siRNA-mediated silencing of *MALAT-1* resulted in a decrease of the EMT-associated ZEB1, ZEB2 and Slug levels, and an increase of E-cadherin levels [37]. Wnt signaling appears to be a core pathway targeted by lncRNAs leading to tumorigenesis (Figure 2A).

Autophagy is activated in cancer cells and contributes to tumor cell survival. Interestingly, MEG3 lncRNA likely regulates autophagy because there is a significant negative correlation between MEG3 levels and the level of an autophagy marker LC3-II in vivo. Moreover, the over-expression of MEG3 markedly suppressed the activation of autophagy and increased apoptosis, whereas knockdown of MEG3 activated autophagy and increased cell proliferation in human bladder cancer cell lines [38]. More importantly, inhibition of autophagy abrogated MEG3 knockdown-induced cell proliferation [38]. Therefore, activation of autophagy and an increase in cell proliferation is the underlying mechanism of aberrant MEG3 expression in bladder cancer.

### Application of lncRNA in bladder cancer

When they are taken together, lncRNAs promote urothelial cell proliferation and suppress cellular apoptosis along with well-defined, hallmark signaling pathways leading to malignant transformation (Figure 2A). As our understanding of the molecular pathways in urothelial oncogenesis expands, reliable biomarkers of bladder cancer are urgently needed. Dysregulated lncRNAs in bladder cancer could become the biomarkers for both diagnosis and prognosis of bladder cancer. NcRNAs are relative stable in cells present in urine and the differential expression of mitochondrial non-coding RNAs (sense and antisense) in cells isolated from voided urine of patients with bladder cancer was recently used as a noninvasive diagnostic assay [43]. A pilot study took advantage of this to evaluate the potential application of UCA1 in urinary sediments from patients with bladder cancer. It turned out to be especially valuable for superficial G(2)-G(3) patients at a high risk for muscular invasion and the sensitivity was 86.4% and 92.3%, respectively, indicating UCA1 is an another new promising urinary marker for the diagnosis of bladder cancer [44]. A lncRNA H19 with oncogenic properties is upregulated in a wide range of tumors including bladder cancer and it is an interesting target of alternative cancer treatment. To utilize the uniqueness of H19 sequence, a plasmid composed of the H19 gene regulatory element that drives the expression of diphtheria toxin (DT-A) gene has been developed and it is undergoing clinical testing as a treatment for superficial bladder cancer and other cancers [45-47]. Direct targeting of dysregulated lncRNAs in bladder cancer is an attractive strategy for alternative treatment, although it is in an infant stage.

### Prospect

Bladder cancer is a common malignant tumor world-wide and survival rate of the invasive subtype remains poor, despite therapeutic advances. Understanding the defect in the gene regulatory network at the genomic level is urgently needed. Recently, thousands of lncRNAs have been identified and disease-associated lncRNA profiles, obtained with a variety of molecular approaches, have placed lncRNAs on the stage of integrated cancer biology. Functional studies have indicated that some lncRNAs are involved in human cancer pathogenesis, acting as either oncogenes or tumor suppressors. A handful of bladder-cancer-related lncRNAs play essential biological roles in tumor development and metastasis, and they provide an opportunity to develop novel biomarkers for bladder cancer diagnosis and a potential for targeted therapy. The biology of lncRNAs is opening a new avenue to unravel causes and develop treatments of bladder cancer, which may yet place lncRNAs at center stage in bladder cancer biology.

## Competing interests

The authors declare no competing interests.

## Authors’ contributions

QNZ and MS conceived this review and drafted the manuscript; GML and JDW supervised and gave final approval of this version to be published. All authors read and approved the final manuscript.
